# Does Salt Form Matter? A Pilot Randomized, Double-Blind, Crossover Pharmacokinetic Comparison of Crystalline and Regular Glucosamine Sulfate in Healthy Volunteers

**DOI:** 10.3390/nu17152491

**Published:** 2025-07-30

**Authors:** Chuck Chang, Afoke Ibi, Yiming Zhang, Min Du, Yoon Seok Roh, Robert O’Brien, Julia Solnier

**Affiliations:** 1 Clinical Research, ISURA, Burnaby, BC V3N 4S9, Canada; aibi@isura.ca (A.I.); yzhang@isura.ca (Y.Z.); mdu@isura.ca (M.D.); kroh@isura.ca (Y.S.R.); jsolnier@isura.ca (J.S.); 2 Biology Department, University of British Columbia, Kelowna, BC V1V 1V7, Canada; r.ob@suprarnd.ca

**Keywords:** glucosamine sulfate, crystalline glucosamine, bioavailability, pharmacokinetics, human study, randomized crossover trial, nutraceuticals, dietary supplements

## Abstract

**Background**: Crystalline glucosamine sulfate (cGS) claims to be a stabilized form of glucosamine sulfate with a defined crystalline structure intended to enhance chemical stability. It is proposed to offer pharmacokinetic advantages over regular glucosamine sulfate (rGS) which is stabilized with potassium or sodium chloride. However, comparative human bioavailability data are limited. Since both forms dissociate in gastric fluid into constituent ions, the impact of cGS formulation on absorption remains uncertain. This pilot study aimed to compare the bioavailability of cGS and rGS using a randomized, double-blind, crossover design. **Methods**: Ten healthy adults received a single 1500 mg oral dose of either cGS or rGS with a 7-day washout between interventions. Capillary blood samples were collected over 24 h. Glucosamine and its metabolite concentrations were quantified by Liquid Chromatography-High Resolution Mass Spectrometry (LC-HRMS), and pharmacokinetic parameters—including maximum concentration (C_max_), time to reach C_max_ (T_max_), and area under the curve (AUC)—were calculated. **Results**: Mean AUC_0–24_, C_max_, T_max_, and T_½_ values for glucosamine and glucosamine-6-sulfate (GlcN-6-S) were comparable between cGS and rGS. Although the AUC_0–24_ for glucosamine was modestly higher with rGS (18,300 ng·h/mL) than with cGS (12,900 ng·h/mL), the difference was not statistically significant (*p* = 0.136). GlcN-6-S exposure was also similar between formulations (rGS: 50,700 ng·h/mL; cGS: 50,600 ng·h/mL), with a geometric mean ratio of 1.39, a delayed T_max_ (6–8 h) and longer half-life, consistent with its role as a downstream metabolite. N-acetylglucosamine levels remained stable, indicating potential homeostatic regulation. **Conclusions**: This pilot study found no significant pharmacokinetic advantage of cGS over rGS. These preliminary findings challenge claims of cGS’ pharmacokinetic superiority, although the small sample size limits definitive conclusions. Larger, adequately powered studies are needed to confirm these results.

## 1. Introduction

Glucosamine sulfate is a naturally occurring amino monosaccharide involved in the biosynthesis of glycosaminoglycans, key structural components of cartilage and synovial fluid [1,2]. It is widely used as a dietary supplement for the management of osteoarthritis, a degenerative joint disease characterized by cartilage breakdown and inflammation [3,4]. The rationale for glucosamine sulfate supplementation stems from its ability to potentially stimulate chondrocyte metabolism, inhibit cartilage degradation, and reduce inflammation [5,6,7,8,9]. Moreover, both short- and long-term supplementation are widely regarded as safe and well tolerated [10,11,12].

**Figure 1:**
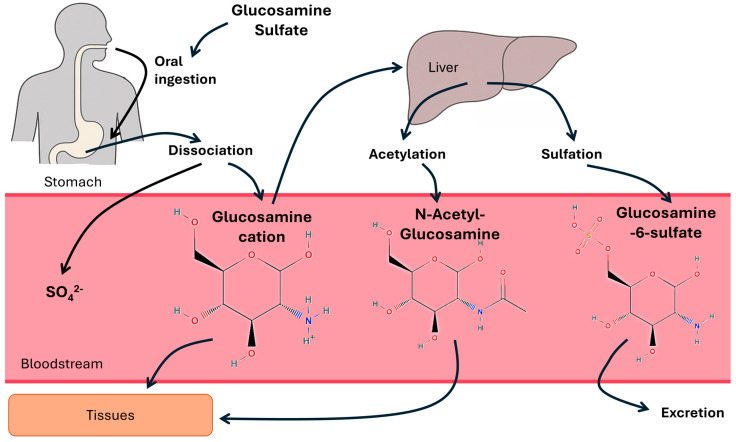
Metabolic process of Glucosamine Sulfate. After oral ingestion and gastric dissolution, glucosamine sulfate yields the glucosamine cation, which is absorbed and distributed. It undergoes acetylation to form N-acetyl glucosamine, sulfation to produce glucosamine 6-sulfate, or enters directly into tissue metabolism. Glucosamine 6-sulfate is excreted.

The bioavailability of glucosamine sulfate is a critical determinant of its therapeutic efficacy [13,14]. After oral administration, the glucosamine sulfate supplement is first dissolved and dissociated in the gastric juices where the glucosamine must be absorbed through the gastrointestinal tract before it enters systemic circulation, where it must be distributed to target tissues, particularly the joint cartilage [15,16]. Once absorbed, glucosamine may undergo intracellular transformations such as phosphorylation and acetylation, forming related derivatives including glucosamine-6-sulfate (GlcN-6-S) and N-acetyl-glucosamine (GlcNAc). A simplified schematic of these metabolic relationships is shown in Figure 1. While glucosamine pharmacokinetics are relatively well characterized, the systemic circulating levels of these related compounds have not been extensively studied in humans. This raises important questions about the broader metabolic fate of glucosamine and its potential downstream biological activity.

Pure Glucosamine Sulfate, in the absence of stabilizing salts, is highly hygroscopic and therefore chemically unstable, making it unsuitable for use in consumer products [17]. To address this, manufacturers commonly stabilize the active substance by complexing it with salts containing chloride ions, enabling commercial viability. Most dietary supplement manufacturers regard such ion assemblies—those with identical stoichiometric ratios of glucosamine to counterions—as equivalent, given that these complexes dissociate to yield the same active glucosamine moiety in vivo [18]. This perspective is further supported by in vitro studies showing that glucosamine stimulates production of hyaluronic acid and glycosaminoglycans and exerts consistent anti-inflammatory effects on chondrocytes and synovial cells, including inhibition of NF-κB activation and prostaglandin E_2_ synthesis [19]. Among these formulations, the patented crystalline glucosamine sulfate (cGS) has been proposed to exhibit superior pharmacological activity compared to regular glucosamine sulfate (rGS) [20].

While cGS is only stabilized with sodium chloride, regular glucosamine sulfate (rGS), is typically stabilized with potassium chloride or sodium chloride, and contains an assembly of ions in a ratio that is the same as the patented cGS. For consumers, the distinction between these forms is further obscured by regulatory labeling requirements. For example, Health Canada allows products to be labeled simply as “Glucosamine Sulfate” on the front panel even when the actual source materials could be either “Glucosamine Sulfate Sodium Chloride” or “Glucosamine Sulfate Potassium Chloride” [21]. Regulations in several other countries are similarly ambiguous.

Furthermore, cGS and rGS appear to exhibit similar pharmacological activity. Studies on cGS have shown its ability to inhibit proinflammatory mediators in human osteoarthritic chondrocytes and to attenuate NF-κB activation [22,23]. Comparable anti-inflammatory effects have also been reported for rGS, including evidence of active transport into human chondrocytes and suppression of NF-κB in rat models [24,25]. While several pharmacokinetic studies have examined the bioavailability of glucosamine sulfate in humans [13,26,27,28,29], only one prior study has directly compared the bioavailability of cGS and rGS in a crossover design [30]. However, that study employed a charged aerosol detector, which lacked the sensitivity and specificity offered by high-resolution mass spectrometry that also allows for simultaneous measurements of key metabolites.

Since both cGS and newer rGS formulations are widely used in clinical practice, establishing their bioequivalence is essential to support the use of cost-effective alternatives and ensure consistent therapeutic outcomes. Based on our hypothesis that both cGS and rGS dissociate in gastric fluid to yield a common glucosamine cation intermediate, no pharmacokinetic differences between formulations would be expected. Previous findings suggest formulation-dependent differences may simply reflect variation in solvation efficiency rather than fundamental differences in the active moiety. Demonstrating bioequivalence would therefore support the use of rGS as a clinically viable substitute for cGS and provide evidence consistent with this dissociation-based mechanism.

Given the exploratory nature and limited sample size, this pilot study evaluated the bioavailability of cGS and rGS in healthy adults using a randomized, double-blind, crossover design. We hypothesized that cGS would not result in significantly greater systemic exposure to glucosamine, as reflected by key pharmacokinetic (PK) parameters, including area under the curve (AUC), peak plasma concentration (C_max_), and time to maximum concentration (T_max_).

To our knowledge, this is the first human study to simultaneously quantify glucosamine and its two primary metabolites—glucosamine-6-sulfate (GlcN-6-S) and N-acetyl-glucosamine (GlcNAc)—over a 24 h period following oral administration. While glucosamine pharmacokinetics have been previously characterized, circulating levels of GlcN-6-S and GlcNAc have not been systematically assessed offering a more comprehensive view of glucosamine metabolism and potential downstream activity.

## 2. Materials and Methods

### 2.1. Study Design

This pilot study employed a double-blind, randomized, crossover design conducted in accordance with the CONSORT 2010 guidelines and the CONSORT extension for crossover trials [31]. All participants provided written informed consent. Participants received single doses of cGS and rGS, with a 7-day washout period between interventions. The study was managed and conducted at the research facility of ISURA (Burnaby, BC, Canada) between January and March of 2025.

The study protocol was approved by the Institutional Review Board (IRB) of the Canadian Shield Ethics Review Board (REB tracking number: 2022-11-002, with OHRP Registration IORG0003491, FDA Registration IRB00004157) on 1 April 2024. The study was registered at ClinicalTrials.org [trial registry number: NCT06971484 on 22 May 2025].

### 2.2. Participants

**Figure 2:**
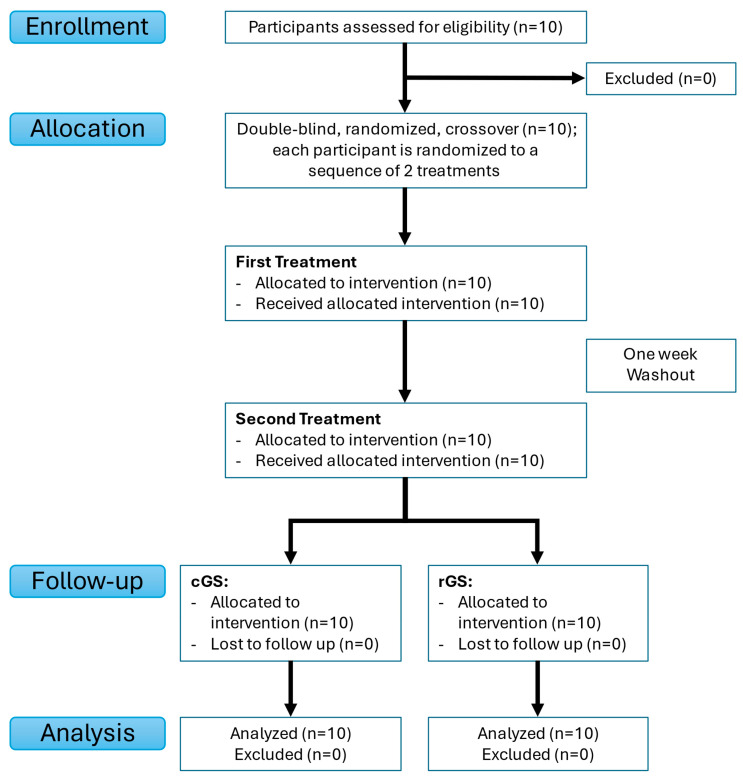
Study flow diagram. Flow diagram of the randomized, double-blind, crossover trial (n = 10). cGS = crystalline glucosamine sulfate. rGS = regular glucosamine sulfate. All participants received both treatments with a one-week washout between phases. No losses to follow-up or exclusions occurred; all participants were included in the final analysis.

This exploratory study was designed as a preliminary investigation to assess pharmacokinetic comparability; therefore, no prior sample size calculation was conducted. Based on feasibility constraints (e.g., recruitment, budget, and analytical capacity) and similar numbers from previous pharmacokinetic studies [25,27], 10 healthy adults (female and male; age range: 21–65 years) with no recent use of glucosamine supplements were recruited and enrolled from the general adult population (ages 21–65) without specific sex stratification, reflecting typical users of glucosamine supplements. Exclusion criteria included a history of gastrointestinal disorders, current pregnancy or lactation, presence of chronic medical conditions such as liver or kidney disease, use of medications or supplements known to affect glucosamine metabolism; known allergies to shellfish or glucosamine-containing products. All participants were non-smokers in generally good health, as assessed by medical history and screening questionnaires. See Figure 2 for the flow diagram of this study.

### 2.3. Interventions

Two commercial products were purchased online as interventions for this study. The two commercial glucosamine sulfate products were selected to reflect formulations commonly available to consumers in North America, based on market availability and consistent product labeling in order to assess systemic glucosamine exposure under conditions representative of real-world use. See Table 1 for their detailed compositions. During each intervention, participants received one of two products according to their randomized sequence with a washout period of at least one week between interventions:

- Regular glucosamine sulfate (rGS): A single dose of 1500 mg of regular glucosamine sulfate.
- Crystalline glucosamine sulfate (cGS): A single dose of 1500 mg of crystalline glucosamine sulfate.

Participants were instructed to fast for 10 h prior to arriving at the research facility between 7:00 and 9:00 a.m. for each intervention to minimize diurnal variability and ensure consistency across sessions. Intervention was administered with a glass (200 mL) of water immediately after collection of the Time 0 blood sample.

**Table 1:** Study interventions.

Intervention	rGS	cGS
Dosage form	Hard-gelatin capsules	Caplets
Number of dosage forms per dose	2	2
Content per dose	1500 mg Glucosamine Sulfate Potassium Chloride	1500 mg Glucosamine Sulfate Sodium Chloride
Disintegration Time (min)	<13 *	<30 *
Source of Glucosamine	Shrimp/crab exoskeleton	Shellfish
Non-medicinal ingredients	Gelatin capsule (gelatin, purified water), magnesium stearate, microcrystalline cellulose	Microcrystalline cellulose, Polyvinyl Pyrrolidone K25, Cross-linked Sodium Carboxymethylcellulose, Hydroxypropyl Methylcellulose, Polyethyleneglycol 6000, Magnesium Stearate, Titanium Dioxide, Talc, Polydextrose FCC, Maltodextrin, Medium chain triglycerides.
Brand	Webber Naturals	Dona (Distributed by WynnPharm Inc.)
Product Name	Joint Ease Glucosamine Sulfate	Crystalline Glucosamine Sulfate
UPC	25273 05052	91108 06010
Lot Number	964851	B2301639
Expiry Date	May 2028	February 2027
Distributor Location	Coquitlam, BC, Canada	Freehold, NJ, USA

rGS: Regular Glucosamine Sulfate, cGS: Crystalline Glucosamine Sulfate; * both products meet USP disintegration requirements.

### 2.4. Randomization and Blinding

Microsoft Excel (2016, Redmond, WA, USA) was used to generate a randomization sequence using its random number function RAND(). Participants were randomized in a 1:1 ratio to receive one of two possible treatment sequences. The randomization sequence was generated by the study coordinator who also allocated the interventions by placing the respective pills into identical, opaque containers, each individually labeled with a unique study code corresponding to the randomization list. Thereafter, the randomization sequence was stored in a secure folder to be opened only in case of emergency. Both participants and study personnel, including investigators and outcome assessors, were blinded to the treatment assignments to ensure allocation concealment.

### 2.5. Blood Sample Collection and Analysis

Blood samples were collected at 0, 0.5, 1, 2, 3, 4, 6, 8, 10, 12, and 24 h after administration of each formulation using EDTA-coated capillaries (Sarstedt, Germany) and immediately frozen at −20 °C. Concentrations of glucosamine in blood were determined according to previously published method using LC-MS [14,32,33]. The method was modified to run on an Orbitrap HRMS instead of a triple-quadrupole mass spectrometer. The modified protocol is detailed below.

On the day of sample preparation, frozen samples were allowed to equilibrate at room temperature prior to processing. Each sample containing 50 µL of whole blood was diluted with 400 µL of the freshly prepared internal standard solution (1:5 dilution). Samples were next centrifuged at 16,000× *g* for 5 min at room temperature and then transferred onto a microplate for LC-MS processing using a ThermoFisher Vanquish UHPLC system (Waltham, MA, USA) coupled to a ThermoFisher Q-Exactive Orbitrap Mass Spectrometer (Waltham, MA, USA). The Orbitrap mass spectrometer was calibrated at 70,000 resolution with an accepted range for mass deviation of ±5.0 ppm using Thermo Pierce LTQ Velos ESI Positive Ion Calibration Solution and Thermo Pierce ESI Negative Ion Calibration Solution. The UHPLC was operated with the following parameters: 0.5% formic acid in water was used as Mobile Phase A, and 0.5% formic acid in acetonitrile was used as B. Liquid chromatography was carried out with an initial isocratic step of 98% A and 2% B for 4 min, followed by a solvent gradient progressing from 2% A to 65% B in 1.5 min. The column was then rinsed with 100% B for 1 min, and the equilibrated at 2% B for 4.0 min before the next injection.

The separation was performed at a flow rate of 400 µL/min on an Acme Xceed C18, 100 mm × 2.1 mm, 1.9 µm column (Phase Analytical Technology, State College, PA, USA). LC-MS grade solvents and formic acid were obtained from Fisher Scientific (Ottawa, ON, Canada). The orbitrap mass spectrometer was operated with positive heated electrospray ionization in Full MS mode with a resolution setting of 70,000, a scan range of 110–1000 *m*/*z*, and a maximum ion trapping time of 200 milliseconds. Glucosamine, ^13^C-glucosamine, glucosamine-6-sulfate, and N-acetyl-glucosamine were detected as their potassium adducts (C_6_H_13_NO_5_·K^+^, *m*/*z* = 218.0425; ^13^CC_5_H_13_NO_5_·K^+^, *m*/*z* = 219.0459; C_6_H_13_NO_8_S·K^+^, *m*/*z* = 297.9994; C_8_H_15_NO_6_·K^+^, *m*/*z* = 260.0531, respectively). D-(+)-glucosamine hydrochloride (≥99%, HPLC) and D-glucosamine-1-^13^C hydrochloride (99 atom% ^13^C) were obtained from Millipore Sigma (Oakville, ON, Canada).

The quantification method was validated for accuracy, precision, and selectivity, with the calibration curve showing good linearity (R^2^ > 0.997) from 0.5 to 100 ng/mL of glucosamine, and the lower limit of quantification (LLOQ) was 0.52 ng/mL.

### 2.6. Outcomes and Pharmacokinetic Analyses

The primary outcome was the result of the pharmacokinetic analysis of blood glucosamine concentrations. Secondary outcomes were results from PK analyses on GlcN-6-S and GlcNAc.

For GlcN-6-S, baseline correction was performed by subtracting the mean of the 0 h and 24 h time points from all time points for each individual. This approach was selected to minimize variability arising from isolated fluctuations and assumes return to baseline by 24 h. The corrected concentrations were used in the calculation of pharmacokinetic parameters and metabolite-to-parent (M/P) ratios.

Pharmacokinetic parameters were determined using non-compartmental analysis (NCA) via the software PKSolver (version 2.0). The following pharmacokinetic parameters were calculated for both formulations: maximum plasma concentration (C_max_), time to reach maximum concentration (T_max_), area under the plasma concentration-time curve (AUC) from 0 to 24 h (AUC_0–24_), and elimination half-life (T_1/2_).

### 2.7. Supplementary Safety Monitoring

To evaluate biochemical tolerability, a supplementary cohort of 12 healthy adults received single doses of both rGS and cGS in a randomized crossover design under the same conditions as the main study. Blood samples were collected at 0 h and 24 h post-dose to measure standard clinical chemistry and electrolyte parameters. Full details and results are provided in Supplementary Tables S1 and S2.

### 2.8. Statistical Analysis

Blood-concentrations-over-time data for GlcN, GlcN-6-S, and GlcNAc between treatments were compared using Repeated Measures Mixed-effects Analysis with Šídák multiple comparisons correction.

Pharmacokinetics data were assessed for normality using the Shapiro-Wilk test. Paired *t*-tests were used for normally distributed data, and the Wilcoxon signed-rank test was applied for non-normally distributed variables. Holm-Šídák correction was applied for multiple comparisons. A two-tailed *p*-value of <0.05 was considered statistically significant. Unless otherwise stated, Confidence Intervals (CI’s) of 95% were reported. For consistency with bioequivalence reporting conventions, GMR’s are also reported with 90% CIs. All analyses were performed using GraphPad Prism (version 10.4.1, Dotmatics, Boston, MA, USA).

Despite the small sample size, a post hoc power analysis indicated that the study had 95.3% power to detect the observed AUC_0–24_ difference between formulations, assuming a realistic within-subject correlation of 0.6. This suggests that the non-significant *p*-value is unlikely due to insufficient power, but rather due to inter-individual variability or conservative statistical thresholds.

## 3. Results

### 3.1. Participant Characteristics

A total of 10 healthy participants (4 males and 6 females) were enrolled (Table 2). The resulting sex distribution (4 males, 6 females) reflects availability of eligible volunteers rather than targeted subgroup quotas. The mean age was 42.3 ± 10.7 years for males and n 34.3 ± 7.4 years for females, with an overall mean of 37.5 ± 9.3 years. The average body mass index (BMI) across all participants was 25.1 ± 2.9 kg/m^2^, which falls within the overweight range based on WHO criteria.

### 3.2. Pharmacokinetics

**Figure 3:**
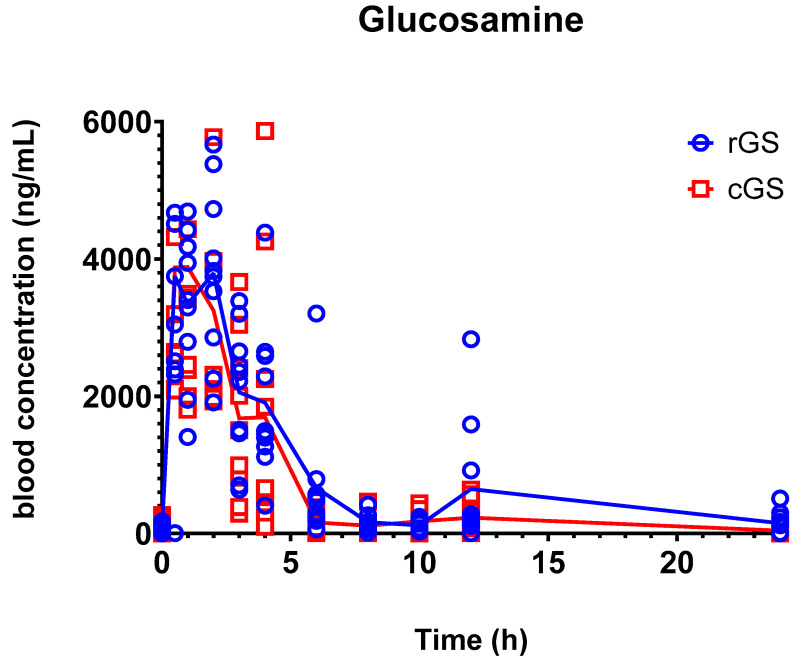
Mean glucosamine concentrations in participants’ whole blood over time after single 1500 mg oral dose of regular glucosamine sulfate (rGS) and crystalline glucosamine sulfate (cGS). Data points represent individual participant concentration with lines indicating mean values for each formulation. No statistically significant differences between the two interventions were found.

As shown in Figure 3 and Table 3, rGS demonstrated higher mean AUC_0–24_ and C_max_ values compared to cGS, with geometric mean ratios (GMRs) of 1.69 and 1.57, respectively. However, these differences did not reach statistical significance (*p* = 0.1360 and 0.652 for AUC_0–24_ and C_max_, respectively), likely due to interindividual variability. The 90% confidence interval for the GMR of AUC_0–24_ (1.19–2.41) exceeded the conventional bioequivalence range of 0.80–1.25, suggesting a potential difference in systemic exposure whereas the lower limit for C_max_ is narrowly below 1.00, which may also indicate a trend toward higher peak concentrations with rGS. Median T_max_ and mean elimination half-lives (T_1/2_) were comparable between formulations.

#### 3.2.1. Glucosamine-6-Sulfate (GlcN-6-S)

**Figure 4:**
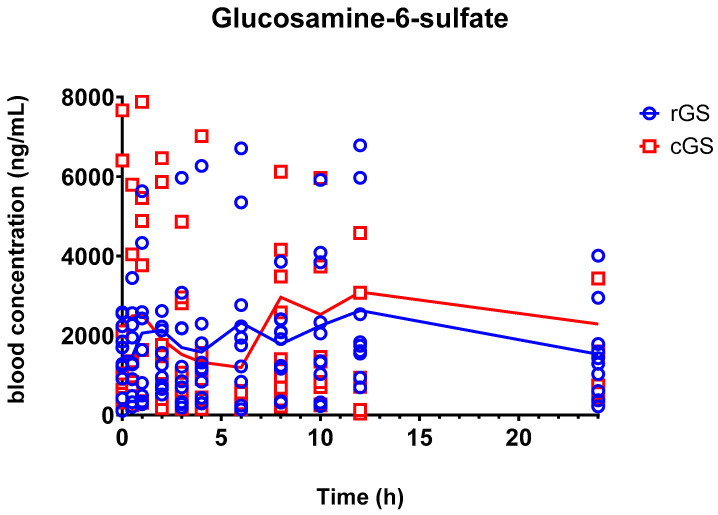
Mean concentrations of glucosamine 6-Sulfate in participants’ whole blood over time. Individual data plotted along the group means. No significant differences between the two interventions were found. Regular glucosamine sulfate (rGS); Crystalline glucosamine sulfate (cGS).

GlcN-6-S exhibited comparable overall exposure between formulations, with similar AUC_0–24_ values but substantial interindividual variability (GMR = 1.39, 90% CI: 0.641–3.02; Table 4). C_max_ was slightly higher with cGS, although this difference did not reach statistical significance (*p* = 0.341). GlcN-6-S exhibited a delayed Tmax, with median values of 6.75 (95% CI: 1.65–11.9) and 7.05 (95% CI: 3.8–10.3) hours for rGS and cGS, respectively (Figure 4, Table 4); it also showed a longer terminal half-life (38.7 h and 43.8 h), suggesting slower elimination.

#### 3.2.2. N-Acetyl-Glucosamine (GlcNAc)

**Figure 5:**
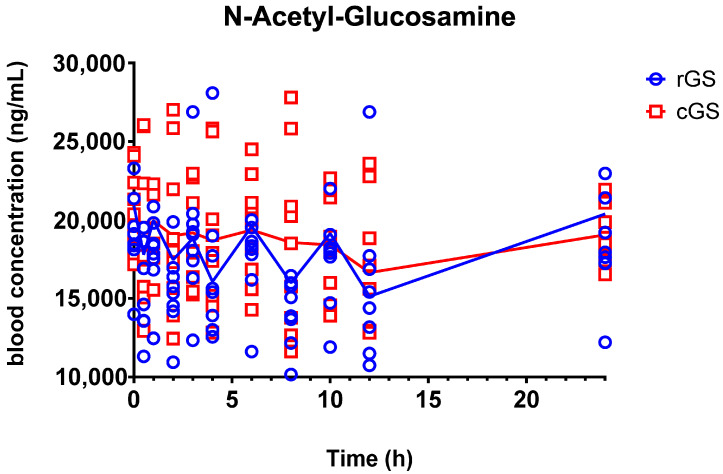
Mean blood concentrations of N-acetyl glucosamine concentrations in participants over time. Individual data plotted along the group means. No significant changes were observed over time when compared against 0 h. Regular glucosamine sulfate (rGS); Crystalline glucosamine sulfate (cGS).

GlcNAc plasma concentrations remained stable throughout the 24 h period in both treatment arms, with no discernible peaks (Figure 5). This suggests that GlcNAc is either homeostatically regulated or not substantially affected by exogenous glucosamine intake. Accordingly, pharmacokinetic parameters and statistical comparisons were not calculated.

#### 3.2.3. Metabolite-to-Parent Ratio Profiles

To further characterize the metabolic conversion of glucosamine, metabolite-to-parent (M/P) ratios were calculated for both C_max_ and AUC_0–24_ using GlcN-6-S as the primary metabolite. As shown in Table 5, the M/P ratios for C_max_ were 0.27 for rGS and 0.54 for cGS. The corresponding AUC_0–24_ M/P ratios were 0.50 and 0.68, respectively (Table 5). Although cGS yielded numerically higher M/P ratios, the differences were not statistically significant (*p* = 0.139 for C_max_ M/P and *p* = 0.179 for AUC_0–24_ M/P), indicating similar metabolic conversion between formulations.

### 3.3. Supplementary Safety Data

Supplementary safety data from a matched cohort of 14 participants revealed no clinically significant changes in blood chemistry or electrolytes following administration of either rGS or cGS. See Supplementary Tables S1 and S2 for full details.

## 4. Discussion

This exploratory pilot study compared the pharmacokinetics of rGS and cGS using a randomized crossover design in healthy volunteers. Blood concentrations of glucosamine (GlcN), glucosamine-6-sulfate (GlcN-6-S), and N-acetyl-glucosamine (GlcNAc) were monitored over a 24 h period post-dose to evaluate absorption and metabolic patterns. Although safety data were not collected in the pharmacokinetic cohort, a supplementary analysis in a parallel group of 12 participants confirmed the biochemical tolerability of both formulations. No laboratory abnormalities or adverse events were observed. While reassuring, these results should be confirmed in larger studies with integrated safety and pharmacokinetic monitoring. (See Supplementary Tables S1 and S2).

Both formulations showed similar overall PK profiles with rGS showing a higher mean C_max_ (6000 ng/mL) than cGS (4160 ng/mL), though variability was substantial (95% CI for cGS: 2080–6240 ng/mL). These values align with prior reports, which also demonstrated broad interindividual variability and method-dependent differences [27,30]. For example, a small crossover study involving 14 participants reported a mean C_max_ of 2125 ng/mL (SD: 369; range: 1516–2766) for cGS and 2224 ng/mL (SD: 354.5; range: 1691–3044) for rGS, using HPLC-CAD [30]. Another study with 26 participants reported C_max_ values of 4.51 µg/mL for cGS and 4.95 µg/mL for glucosamine sulfate potassium chloride, measured via LC-MS/MS, which are in close agreement with the present findings [33].

A secondary glucosamine concentration peak was noted at 12 h post-dose for both formulations, potentially reflecting enterohepatic recirculation or redistribution from tissues such as cartilage. Similar delayed peaks have been reported, including one at 18 h in a study co-administering glucosamine with chondroitin sulfate [27,28]. While we did not extend sampling beyond 24 h, the observed 12 h rise may suggest an early phase of redistribution or hepatic recycling.

### 4.1. Glucosamine Pharmacokinetics and Bioequivalence

Both formulations were rapidly absorbed, with median T_max_ values of 1.20 h for rGS and 0.93 h for cGS, indicating fast systemic availability post-gastric dissolution; hence, no metabolic precursors likely exist prior to the glucosamine cation. Terminal half-lives averaged 8.17 and 6.83 h, respectively, which indicates similar absorption kinetics (Table 3). Although not statistically significant, rGS showed higher systemic exposure, with geometric mean ratios (GMRs) for AUC_0–24_ and C_max_ of 1.69 and 1.57. Notably, the 90% CI for AUC (1.19–2.41) and for C_max_ (0.99–2.48). According to regulatory guidelines, two formulations are considered bioequivalent if the 90% confidence intervals of their GMRs for both AUC and C_max_ fall entirely within the 0.80–1.25 range [34]. Since both parameters exceeded the regulatory bioequivalence range of 0.80–1.25, suggesting the formulations may not be bioequivalent, with rGS trending toward greater system exposure, though results should be interpreted cautiously due to the small sample size and variability. Despite the small sample, a post hoc power analysis (95.3% power assuming r = 0.6) suggests the non-significant result likely reflects inter-individual variability or conservative thresholds—not inadequate power.

Given that previous studies have favored cGS for its proposed pharmacokinetic advantages [20], our data suggest that rGS may, in practice, offer equal or greater bioavailability, possibly due to differences in salt type, excipients, or dissolution characteristics. As previously reported, individual PK variability remain a significant contributor to these observed differences [30,33]. Nevertheless, their similar PK profiles support the hypothesis that both formulations yield the same solvated glucosamine cation, as a common intermediate, in the stomach, which drives systemic availability regardless of salt form or stabilization. While higher plasma glucosamine levels have been associated with symptom relief in osteoarthritis, the clinical relevance remains uncertain, as plasma concentrations may not directly reflect joint tissue levels [10]. Future PK-PD (pharmacodynamic) studies are needed to clarify this relationship.

### 4.2. Metabolite Insights: GlcN-6-S and GlcNAc

**Figure 6:**
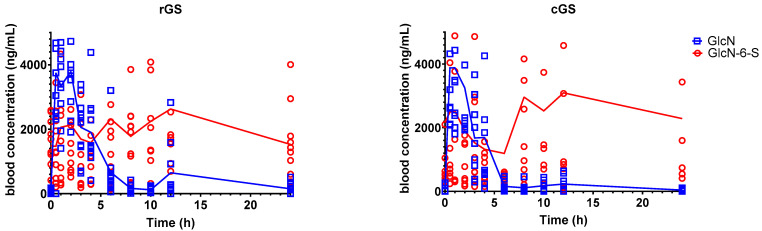
Comparison of blood concentrations of glucosamine and its metabolite over time for both treatments. rGS = regular glucosamine. cGS = crystalline glucosamine sulfate. GlcN = Glucosamine. GlcN-6-S = Glucosamine-6-sulfate.

The delayed peak and prolonged half-life of GlcN-6-S compared to its parent compound support the hypothesis that it is a secondary metabolite formed post-absorption, possibly via hepatic or renal sulfation pathways (Figure 6). This is consistent with prior work suggesting enzymatic conjugation after systemic glucosamine exposure [35].

The observed bimodal concentration-time profile (Figure 6) in some participants suggests the possibility of enterohepatic recirculation or delayed release from tissue reservoirs, as reported in previous PK studies involving chondroitin co-administration or radiolabeled tracers [27,36].

Despite high variability, no significant difference in GlcN-6-S exposure was observed between formulations. The AUC_0–24_ GMR (rGS/cGS) of 1.39 had a wide CI (0.641–3.02), and although cGS showed a higher C_max_, the GMR of 0.703 (90% CI: 0.465–1.063) was not statistically significant. These results align with earlier findings that downstream glucosamine metabolism is generally formulation-independent [13,33]. Similar metabolite-to-parent ratios between cGS and rGS reinforce this interpretation, although the contribution of endogenous GlcN-6-S complicates precise AUC estimation [37,38].

In contrast, plasma levels of N-acetyl-glucosamine (GlcNAc) remained stable over 24 h, fluctuating between 15,000 and 25,000 ng/mL without discernible post-dose peaks (Figure 5). This aligns with prior findings that GlcNAc is not a primary metabolite of oral glucosamine but is instead maintained through tightly regulated endogenous pathways [38,39,40]. While glucosamine can theoretically enter the hexosamine biosynthetic pathway, its contribution to systemic GlcNAc appears minimal at standard doses [24], likely due to homeostatic mechanisms linked to protein glycosylation and O-GlcNAc signaling [41,42].

### 4.3. Metabolite-to-Parent Ratios

Metabolite-to-parent (M/P) ratios based on AUC and C_max_ further highlight the metabolic relationship between GlcN and GlcN-6-S. The AUC M/P ratio was approximately 0.5 (rGS: 0.50, 95% CI: 0.20–1.30; cGS: 0.68, 95% CI: 0.23–1.99), while the C_max_ M/P ratio was around 0.4 (rGS: 0.27, 95% CI: 0.13–0.56; cGS: 0.54, 95% CI: 0.23–1.26) (Table 5), suggesting that the extent of exposure to the metabolite is roughly half that of the parent compound, with proportionally lower peak concentrations. These ratios were consistent across formulations, indicating similar metabolic handling of glucosamine irrespective of the formulation used [43]. To our knowledge, no prior studies have reported M/P ratios for GlcN-6-S in humans following oral glucosamine administration, making these findings novel. These values provide preliminary insight into the extent of sulfate conjugation In Vivo and may serve as a basis for future investigations of glucosamine metabolism.

For GlcN-6-S, baseline correction was performed by subtracting the mean of the 0 h and 24 h time points from all time points for each individual. This approach was selected to minimize variability arising from isolated fluctuations and assumes return to baseline by 24 h. While this method cannot fully account for intra-day fluctuations, it reduces the influence of single-point outliers and aligns with established practices for analyzing endogenous metabolites. The corrected concentrations were used in the calculation of pharmacokinetic parameters and M/P ratios. Such M/P ratios are valuable in understanding the pharmacokinetics of metabolite formation, especially for compounds like glucosamine that have known endogenous pools and complex biotransformation pathways [38]. However, interpreting these ratios must be performed cautiously given the presence of baseline endogenous GlcN-6-S, which complicates AUC calculations and may result in apparent negative values after baseline subtraction.

### 4.4. Clinical and Research Implications

The comparable bioavailability of cGS and rGS suggests that formulation type may not meaningfully impact systemic exposure. While this challenges assumptions about the superiority of stabilized forms and supports rGS as a potentially more cost-effective option these findings should be interpreted with caution given the limited sample size. Larger, adequately powered studies are needed to confirm these observations.

For researchers, the similar PK profiles of glucosamine and GlcN-6-S across formulations support cross-study comparability when gastric dissociation yields the same glucosamine cation. The stable GlcNAc levels underscore the importance of accounting for endogenous baselines. Together, these findings reinforce the value of integrated PK/PD studies to clarify exposure-response relationships.

While our findings suggest comparable systemic exposure between cGS and rGS, the clinical implications of these pharmacokinetic results remain uncertain. Glucosamine’s therapeutic effects—particularly in osteoarthritis—are likely influenced by multiple factors beyond plasma concentration, including tissue distribution, cartilage penetration, and local pharmacodynamics. Future studies should incorporate pharmacodynamic (PD) endpoints, such as inflammatory biomarkers or clinical symptom scores, to clarify the relationship between systemic glucosamine exposure and therapeutic benefit.

### 4.5. Limitations

This study has several limitations. The sample size, while adequate for exploratory PK comparisons, limits the precision of bioequivalence estimates. Although a post hoc power analysis indicated >95% power to detect the observed AUC_0–24_ difference between formulations (assuming a within-subject correlation of 0.6), such retrospective analyses cannot replace formal a priori sample size planning. It remains possible that larger studies could detect statistically significant differences not observed here; however, given the substantial inter-individual variability also seen in prior studies, such differences may be of limited clinical relevance. The relatively high inter-individual variability, particularly for GlcN-6-S, further affects the width of confidence intervals. In addition, the endogenous nature of GlcNAc and GlcN-6-S introduces complexity in interpreting baseline-corrected data, which may obscure small but real changes due to treatment.

Also, the single-dose design employed in this study limits the ability to extrapolate results to the steady-state conditions typically seen with long-term glucosamine supplementation. Such chronic dosing may alter pharmacokinetics through cumulative distribution, adaptive metabolic changes, or time-dependent modulation of absorption or clearance pathways. Safety data were derived from a separate cohort rather than the main pharmacokinetic population. Although reassuring, these findings should be interpreted with caution. Therefore, further studies with larger sample sizes employing multiple-dose regimens and extended monitoring are needed to evaluate steady-state pharmacokinetics and long-term formulation equivalence.

## 5. Conclusions

This crossover study found no significant difference in glucosamine absorption between crystalline and regular (non-crystalline) glucosamine sulfate, despite slightly higher exposure with rGS. Both forms yielded similar levels of the major metabolite GlcN-6-S, while GlcNAc levels remained largely unchanged. These findings suggest that neither salt form nor stabilization significantly impacts systemic bioavailability, though this conclusion should be interpreted cautiously given the limited sample size. To our knowledge, this is the first human study to track glucosamine and its key metabolites over 24 h—providing new insights into its metabolic fate and informing future research on clinical relevance.

## Figures and Tables

**Table 2:** Baseline characteristics of the participants.

	Males	Females	Combined
N	4 (40%)	6 (60%)	10 (100%)
Age	42.3 ± 10.7	34.3 ± 7.4	37.5 ± 9.3
Weight (kg)	77.5 ± 9.7	66.8 ± 12.9	71.1 ± 12.4
Height (cm)	171.5 ± 5.8	165.3 ± 6.2	167.8 ± 6.5
BMI (kg/m^2^)	26.3 ± 2.8	24.2 ± 2.8	25.1 ± 2.9

**Table 3:** Pharmacokinetics parameters for glucosamine in study participants.

Parameter	rGS	cGS	*p*-Value	GMR (rGS/cGS)	90% CI
AUC_0–24_ (ng∙h/mL)	18,300 (15,000–21,700)	12,900 (8740–17,200)	0.136	1.69	1.19–2.41
C_max_ (ng/mL)	6000 (3500–8480)	4160 (2080–6240)	0.652	1.57	0.99–2.48
T_max_ (h)	1.20 (0.69–1.71)	0.93 (0.07–1.79)	0.652		
T_1/2_ (h)	8.17 (4.6–11.7)	6.83 (0.86–12.8)	0.652		

Means (95% CI) reported. GMR = Geometric Mean Ratio. GMR is reported with 90% CI at the right-most column for comparison. No statistical significance was found between treatments (Multiple Wilcoxon tests for T_1/2_, T_max_, and C_max_, and multiple paired *t*-tests for AUC_0–24_; Holm-Šídák correction for multiple comparisons was applied).

**Table 4:** Pharmacokinetic parameters for glucosamine-6-sulfate in study participants.

Parameter	rGS	cGS	*p*-Value	GMR (rGS/cGS)	90% CI
AUC_0–24_ (ng∙h/mL)	50,700 (27,500–73,900)	50,600 (8730–92,500)	0.795	1.39	0.641–3.02
C_max_ (ng/mL)	3900 (2100–5700)	5430 (3470–7390)	0.341	0.703	0.465–1.063
T_max_ (h)	6.75 (1.65–11.9)	7.05 (3.8–10.3)	0.795		
T_1/2_(h)	38.7 (10–64.5)	43.8 (−28.5–116)	>0.999		

Means (95% CI) reported. GMR = Geometric Mean Ratio. GMR is reported with 90% CI on the right-most column for comparison. No statistical significance was found between treatments (Multiple Wilcoxon tests for AUC_0–24_ and T_max_, and multiple paired *t*-tests for T_1/2_ and C_max_ AUC_0–24_; Holm-Šídák correction for multiple comparisons was applied).

**Table 5:** Metabolite-to-Parent (M/P) Ratios for C_max_ and AUC_0–24_ Following Oral Administration of rGS and cGS.

	rGS	cGS	*p*-Value
C_max_ M/P	0.27 (0.13–0.558)	0.54 (0.23–1.26)	0.139
AUC_0–24_ M/P	0.50 (0.20–1.30)	0.68 (0.23–1.99)	0.179

Geometric Mean with 95% CI presented. *p*-values calculated with paired *t*-tests and Holm-Šídák correction for multiple comparisons.

## Data Availability

The data are not publicly available due to privacy and ethical restrictions imposed by the study’s informed consent protocol. Data and/or statistical analyses are available upon request subject to IRB approval.

## Supplementary Materials

The following supporting information can be downloaded at https://www.mdpi.com/article/10.3390/nu17152491/s1, Table S1: Clinical chemistry and electrolyte parameters at baseline (0 h) and 24 h post-dose following administration of rGS (n = 14); Table S2: Clinical chemistry and electrolyte parameters at baseline (0 h) and 24 h post-dose following administration of cGS (n = 14).

## Abbreviations

The following abbreviations are used in this manuscript:

GlcN-6-S	Glucosamine-6-sulfate
GlcNAc	N-acetyl-glucosamine
cGS	Crystalline glucosamine sulfate
rGS	Regular glucosamine
PK	Pharmacokinetic
AUC	Area under the curve
C_max_	Peak plasma concentration
T_max_	Time to maximum concentration
T_½_	Elimination half-life
IRB	Institutional Review Board
CI	Confidence Interval
GMR	Geometric Mean Ratio
M/P	Metabolite-to-parent ratio
