# Supplementary material for: Does Salt Form Matter? A Pilot Randomized, Double-Blind, Crossover Pharmacokinetic Comparison of Crystalline and Regular Glucosamine Sulfate in Healthy Volunteers
Source: Nutrients. 2025 Jul 30;17(15):2491. doi: 10.3390/nu17152491 (PMC12348100; doi:10.3390/nu17152491)
Supplement: Supplementary file 1 [file nutrients-17-02491-s001.zip › nutrients-3753192-supplementary.pdf]

Supplementary Materials

Table S1: Clinical chemistry and electrolyte parameters at baseline (0 h) and 24 h post-dose following administration of rGS (n = 14)

	0 h	24 h	p-value	Normal Range
Total Bilirubin (umol/L)	12.5 ± 5.6	12.1 ± 9.2	>0.999999	3.4–21.0
AST (U/L)	24.3 ± 9.7	26.7 ± 12	>0.999999	15–40
ALT (U/L)	25 ± 13	24.5 ± 12	>0.999999	9–50
Crea (umol/L)	67.6 ± 15	64.1 ± 17	>0.999999	44.0–97.0
BUN (mmol/L)	4.46 ± 0.89	4.98 ± 1.5	>0.999999	2.50–8.20
HDL (mmol/L)	1.62 ± 0.25	1.64 ± 0.27	>0.999999	1.16–1.42
LDL (mmol/L)	3.04 ± 1.1	3.07 ± 1	>0.999999	0.50–3.14
TC (mmol/L)	5.08 ± 1.2	5.21 ± 1.2	>0.999999	0.00–5.17
TG (mmol/L)	0.989 ± 0.45	1.12 ± 0.68	>0.999999	0.00–1.70
GLU (mmol/L)	3.79 ± 1.2	3.82 ± 0.82	>0.999999	3.89–6.11
tCO2 (mmol/L)	25.4 ± 0.86	25.1 ± 0.82	>0.999999	22.0–29.0
Na+ (mmol/L)	138 ± 1.2	137 ± 1.5	>0.999999	135.0–147.0
K+ (mmol/L)	4.88 ± 0.4	4.96 ± 0.65	>0.999999	3.40–5.30
Ca (mmol/L)	2.47 ± 0.11	2.43 ± 0.2	>0.999999	2.00–2.58
Cl- (mmol/L)	104 ± 0.93	105 ± 0.79	>0.999999	99.0–112.0

Data are presented as mean ± standard deviation (SD). Paired t-tests were used to compare baseline (0 h) and 24 h post-dose values, with *p*-values adjusted for multiple comparisons using the Bonferroni-Dunn correction. No statistically significant differences were observed in any parameter. All values remained within or close to standard clinical reference ranges, indicating no adverse safety concerns within the 24-hour period. A *p*-value of <0.05 was considered statistically significant.

Table S2: Clinical chemistry and electrolyte parameters at baseline (0 h) and 24 h post-dose following administration of cGS (n = 14)

	0h	24h	p-value	Normal Range
TB (umol/L)	11.6 ± 6.2	12.6 ± 5.6	>0.999999	3.4–21.0
AST (U/L)	23.8 ± 8.2	21.8 ± 6.2	>0.999999	15–40
ALT (U/L)	24.1 ± 12	25.9 ± 13	>0.999999	9–50
Crea (umol/L)	65.7 ± 15	67 ± 14	>0.999999	44.0–97.0
BUN (mmol/L)	4.62 ± 1.3	4.3 ± 0.91	>0.999999	2.50–8.20
HDL (mmol/L)	1.64 ± 0.26	1.59 ± 0.27	0.821466	1.16–1.42
LDL (mmol/L)	3.15 ± 1.1	3.1 ± 1.1	>0.999999	0.50–3.14
TC (mmol/L)	5.36 ± 1.2	5.12 ± 1.2	>0.999999	0.00–5.17

TG (mmol/L)	1.25 ± 0.7	1.01 ± 0.54	>0.999999	0.00–1.70
GLU (mmol/L)	4.06 ± 0.8	3.73 ± 1.1	>0.999999	3.89–6.11
tCO2 (mmol/L)	25.3 ± 0.88	25.5 ± 0.93	>0.999999	22.0–29.0
Na+ (mmol/L)	137 ± 1.7	138 ± 1.2	0.888405	135.0–147.0
K+ (mmol/L)	4.84 ± 0.36	4.93 ± 0.38	>0.999999	3.40–5.30
Ca (mmol/L)	2.48 ± 0.1	2.48 ± 0.1	>0.999999	2.00–2.58
Cl- (mmol/L)	104 ± 1.1	104 ± 0.96	>0.999999	99.0–112.0

Data are presented as mean ± standard deviation (SD). Paired t-tests were used to compare baseline (0 h) and 24 h post-dose values, with *p*-values adjusted for multiple comparisons using the Bonferroni-Dunn correction. No statistically significant differences were observed in any parameter. All values remained within or close to standard clinical reference ranges, indicating no adverse safety concerns within the 24-hour period. A *p*-value of <0.05 was considered statistically significant.
